# Experimental investigation on the effect of nano-silica on reinforced concrete Beam-column connection subjected to Cyclic Loading

**DOI:** 10.1038/s41598-023-43882-5

**Published:** 2023-10-13

**Authors:** G. Shyamala, B. Hemalatha, Yuvarajan Devarajan, Chairma Lakshmi, Dinesh Babu Munuswamy, Nandagopal Kaliappan

**Affiliations:** 1 Department of Civil Engineering, SR University, Warangal, India; 2 Department of Civil Engineering, St Peter’s Institute of Higher Education and Research, Chennai, Tamilnadu India; 3 Department of Mechanical Engineering, Saveetha School of Engineering, SIMATS, Saveetha University, Chennai, Tamilnadu India; 4 Department of Electronics and Instrumentation Engineering, R.M.K. Engineering College, Chennai, Tamilnadu India; 5 Department of Mechanical Engineering, Rajalakshmi Institute of Technology, Chembarambakkam, Chennai, 600124 Tamil Nadu India; 6 Department of Mechanical Engineering, Haramaya Institute of Technology, Haramaya University, Dire Dawa, Ethiopia

**Keywords:** Engineering, Civil engineering

## Abstract

Beam-column joints are crucial load transmission zones because they face concentrated forces from both the beams and the columns. High shear and axial stresses caused by these concentrated forces in the area of the joint may result in decreased joint strength. This article proposes a new beam-to-column connection developed for precast concrete-resisting frames. Concrete mixtures are enhanced mechanically by adding nano silica as it increases compressive strength, flexural strength, and abrasion resistance. Within the concrete, it creates a solid, gel-like matrix that fills voids and strengthens the whole construction. In this study, three reinforced concrete beam-column joint specimens were cast with fly ash, the other three with nano-silica and fly ash, and one sample with nano-silica and a control mix without admixtures was cast. Specimen cast using fly ash and nano-silica is subjected to cyclic loading after 28 days of curing. A load capacity of 100 kN was imposed on the column during testing. It was observed that a gradual increase in fly ash decreased the compressive and flexural strength of the beam-column joints. This decrease in strength was addressed by adding 2.5% nano-silica. Nano silica acts as a nucleus to bond tightly with cement particles during hydration. The results showed that the flexural strength equivalent to that of a controlled specimen can be achieved by adding nano-silica at 2.5% and fly ash at 60%. The highest loading of 38.1 kN can be applied to the specimen with nano-silica without fly ash. Although a higher axial compression ratio can improve the bearing capacity and initial stiffness, it can also reduce deformation capacity and flexibility.

## Introduction

Carbon dioxide release is one reason for global warming, which can be controlled by reducing the huge volume of cement in the construction industry^1^. Efficient waste management uses fly ash to find its disposal in land or water resources. Hence cement is partial replacement by fly ash in concrete by a certain percentage seems to provide the desired strength, but adding fly ash decreases the early strength of concrete^2^. Most of the research works focusing on applying nanomaterials to cementitious composites explored the performance of the materials in various loading/environmental conditions. Possible materials that are currently available with particle sizes below 300 nm includes silica fume and limestone processed materials. By replacement of 10% Silica fume is taken as optimum, it provides maximum compressive strength^3^.

The inclusion of different kinds of nanomaterials to make concrete-based nanocomposites permits novel properties in materials. For example, the capacity to absorb air pollutants, concrete retains more carbon dioxide or self-healing material over time^4^. Nano-silica performs in lowering the corrosion rate of steel-reinforced concrete^5^. Combining nano-silica and micro silica recently induced a synergic effect and increased concrete's strength and durability properties. The research signifies that the nano-silica demands higher super plasticiser usage when compared to micro silica^6^. To achieve the specific properties of high-strength concrete, such as workability, high strength and durability throughout its lifetime, nano-silica and copper slag can be used with the limiting water cement ratio between 0.25 and 0.50^7^. Adding about 2% nano-silica increased the compressive strength of about 17–18% at different testing ages^8^. The capacity of the structure to endure a seismic impact depends to an enormous degree on its capacity to disseminate the energy.

Types of energy dispersal incorporate hysteretic damping, kinetic energy, and viscous damping. In the strong earthquake-prone zone, framed structures should be designed considering earthquake resistance^9^. The durability properties of recycled aggregate can be enhanced by treating the aggregates in nano-silica solution for 1 h, as it improves the surface of microhardness and increases the penetration depth of nano-silica^10^.

The interaction between coarse aggregate and nano-silica in cementitious composites can be described as complimentary. Coarse aggregates serve the purpose of providing structural support, but they may have the drawback of reducing the modulus of elasticity. Conversely, the utilisation of nano-silica, in the absence of coarse aggregates, has the potential to elevate the modulus of elasticity and improve the overall performance of the material. The selection and ratios of these constituents in a concrete mixture will vary based on the desired characteristics of the composite and its intended use^11^. By conducting freeze-soak-sour cycles of a different concrete mix containing nano hydrophobic silane, silica improves durability Concrete workability, strength and durability can be improved using nano-silica in the concrete mix^12^. Nano silica promotes a pozzolanic reaction with early strength. In the end, the quick setting effect of nano silica addition was also identified.

In particular, micro-silica and nano-silica work at two levels; first, the chemical effect of nano-silica and physical micro-silica particles can fill concrete voids. Few researchers reported that adding 1 kg of micro-silica reduces the usage of around 3 kg of cement. Adding nano-silica to cementitious material elevates the viscosity and fills the voids between the cement particles. This greatly impacts the production of Calcium Silicate Hydrate, which influences all mechanical and strength properties of concrete. It also improves cement hydration with a greater surface area of nanoparticles^13^.

The addition of nano-silica increases the density of the cement mixture, water absorption is reduced, and a decrease in the number of voids of recycled aggregate was noticed. In addition, the study's results expose that nano-silica has no considerable outcome on the young modulus of concrete^14, 15^. The molecular structure of a material is modified by adding nano-silica creating special properties in concrete like increasing quality, longevity, strength, and weight reduction can be achieved^16^. The incorporation of nano-silica increases the durability properties of concrete. The addition of nano-silica improves the durability of concrete, so it is suitable for most concrete projects. Even though adding nano-silica elevates the project's construction cost, it can be used for massive construction work like high-storied buildings, bridges, dams, etc.,^17^.

From the experimental works, mixture with the addition of nano-silica had good rusting resistance and fatigue performance even after long term exposure^18^. In self-compacting concrete, the incorporation of nano-silica decreased the fluidity and increased viscosity of the concrete due to minimum particle size and large surface area^19^. It was experienced in the trial mix that the greater quantity of nano-silica demanded a greater quantity of superplasticiser. Early-stage compressive strength attainment was observed by adding nano-silica^20^. The precast beam and column connection method makes it simple by using projecting bars from the beam smaller in diameter than those protruding from the column^21^.

The beam-column joint is critical in a reinforced concrete moment-resisting frame. It is subjected to large forces during severe ground shaking, and its behaviour is affected within the joint; tremendous shear forces develop. The failure to shear is constantly fragile by nature, which is not a structurally acceptable condition. So, it is especially vital to decrease the weakness of the Beam-Column joint.

The beam-column joint in Reinforced Concrete structures is the most significant region to transmit loads from beam to column^22^. The monolithic beam-column joint is most efficient than the non-monolithic beam-column joint^23^. The beam-column joints have constrained load-resisting capacity. At the point when heavy loads are applied during the hour of seismic tremors, the damages are Severe.

Repairing the damages in the joint is tedious; thus, the design should be made by considering the earthquake effect to avoid this. The practical impact of the earthquake on existing beam-column joints in structures is vulnerable. So sufficiently, it must be strengthened. The addition of nano-silica in concrete does the method of improving the strength of concrete structural members. Joint shear failure influences frames' strength, ductility, and stability, focusing on joint shear behaviour^24^. Incorporating longitudinal bars and transversal Reinforcement in the beam-column hinge is required. The extra Reinforcement will reduce cracks, give higher energy scattering, and improve strength and stiffness^25^.

Shear panel elements are used to mimic strength and stiffness loss as a result of joint panel failure. Four nodes where one-dimensional beam and column elements intersect define joints. A joint rotational spring characteristic is presented to account for joint shear panel deformation^26^. It is assumed that the computed moment–curvature response of the beam-column cross-section defines the envelope to the moment-rotation response of the plastic hinge. An assumed plastic-hinge length equal to half the depth of the beam-column element and the plastic hinge's hysteretic response is represented by the previously presented general one-dimensional hysteretic response model with load-path parameters^27^.

Considering the previous studies, beam-column joints experience great inelastic shear distortions with the durable column-weak beam design philosophy. The fragile joint shear collapse of beam-column joints will appreciably decrease the flexibility of structures and consequence in a hazardous failure mechanism. Thus the interior of the beam-column joint should have sufficient shear strength to oppose the high shear forces developed during earthquake attacks^28, 29^. Considering the loading pattern responsible for the failure by excessive rotation at the position of the plastic hinging concerning the failure modes, plastic hinges at the beams close to the column faces in the joints mainly fail by crushing under the compression^30^.

Based on the inference from previous studies, it is found that Nano-silica is an innovative nanomaterial that has shown promise in improving the properties of concrete. It is incorporated into concrete mixtures for beam-column connections is a novel approach that explores the potential benefits of nanotechnology in enhancing the performance of critical structural elements. In addition, the application of cyclic loading in this context is significant. Beam-column connections in buildings and structures often experience cyclic loading due to factors like seismic activity, wind, and dynamic loads. Investigating how nano-silica affects the behavior of these connections under cyclic loading conditions is a specialized and critical aspect of structural engineering research. Hence, the chief objective of study is to determine the effects of nano-silica on reinforced concrete beam-column connections subjected to cyclic loading and to investigate and understand how the incorporation of nano-silica into concrete mixtures can impact the performance and behavior of these critical structural elements.

## Materials

The cementitious material used for the study is ordinary Portland cement of 53 grade complies with IS 269:1989. Portland cement is a highly prevalent construction material that is extensively utilized on a global scale. The classification of cement is determined by its compressive strength, resulting in several grades. Among these categories, 53-grade cement is widely used. Cement with a grade of 53 is renowned for its exceptional compressive strength. The material exhibits enhanced load-bearing capacity and is well-suited for use in scenarios where the preservation of structural integrity is of paramount importance. In comparison to cements of lesser grades, it often exhibits a shorter period of time required for the setting process to occur. Grade 53 cement exhibits a high level of durability, rendering it capable of enduring adverse climatic conditions. Consequently, it is well-suited for outdoor applications and constructions that are subjected to the weather. Cement with a 53-grade designation typically exhibits reduced shrinkage characteristics, hence contributing to the mitigation of cracks in concrete constructions.

**Figure 1:**
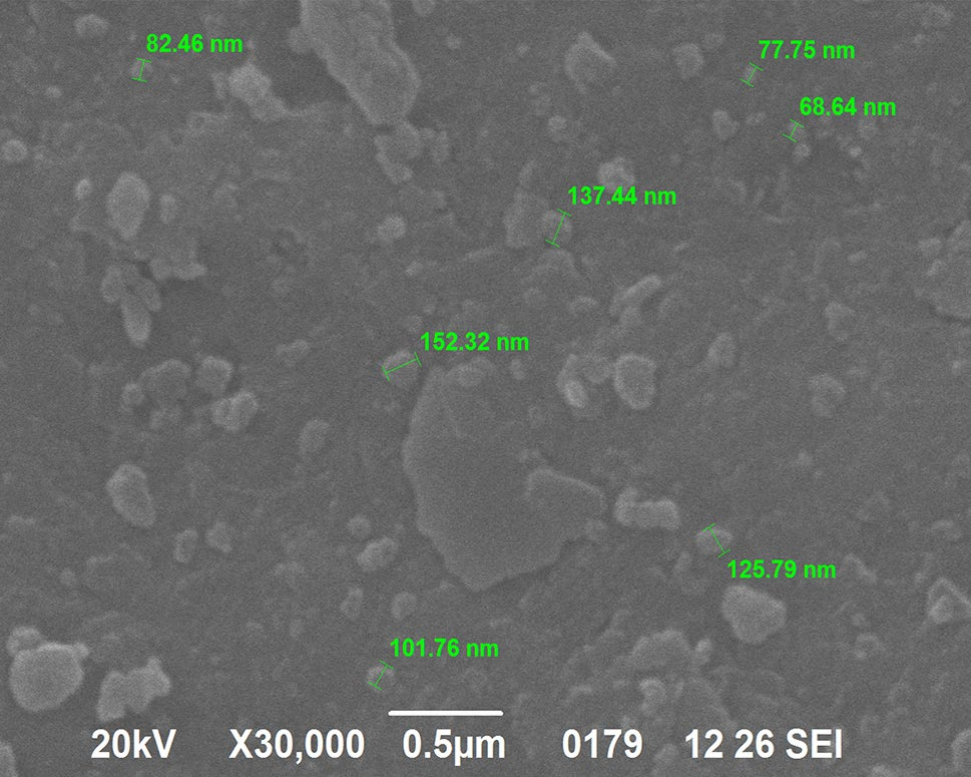
SEM image of nano silica.

**Figure 2:**
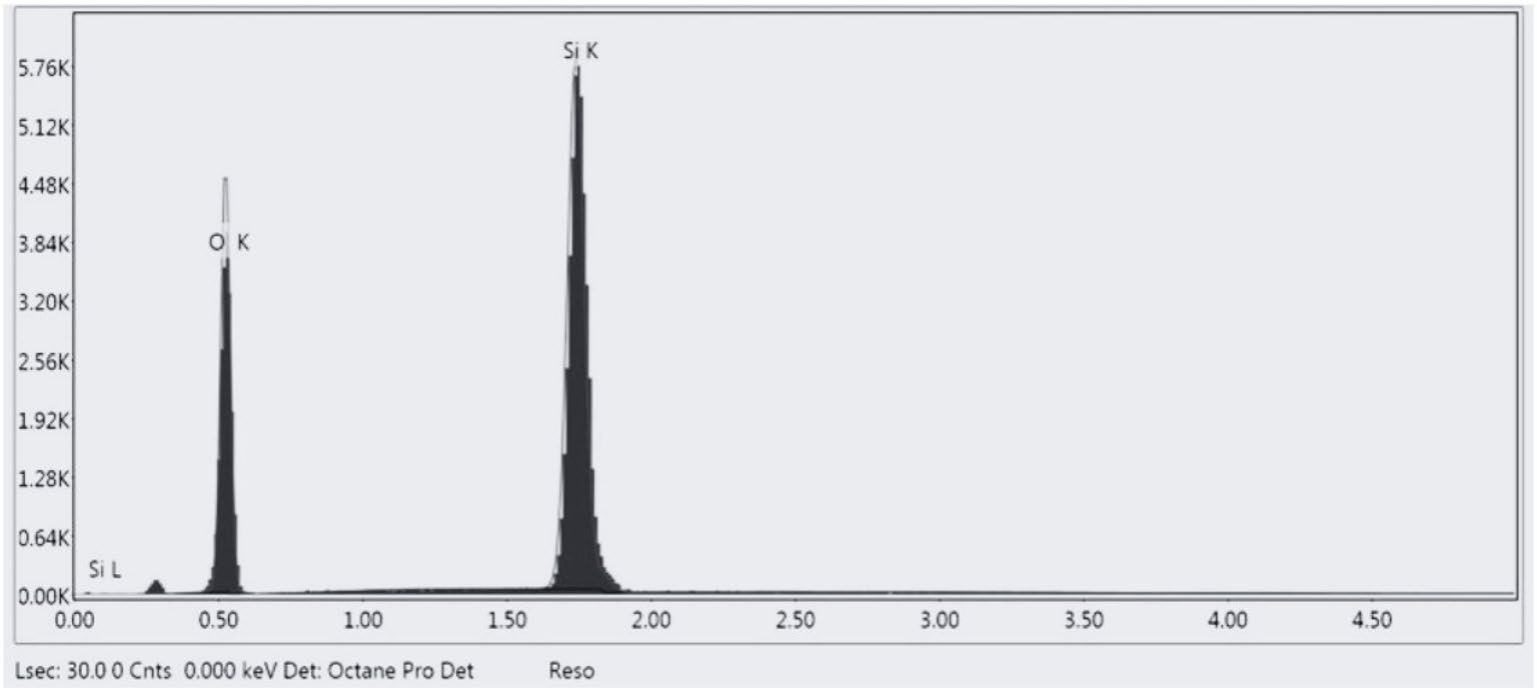
EDX analysis of nano silica.

Natural river sand is used as fine aggregate with a maximum size of 5 mm, a moisture content of 0.11%, and a relative density of 2.65. The coarse aggregate used for the study is with a water absorption of 1.05% of relative density 2.69 with a particle size distribution of 4.75–25 mm. In the ball mill, sand is machined to nano size with a high proportion of silica. The maximum size of the nano-silica range is 152 nm, and the minimum nano-silica particle size range is 68 nm, so 107 nm was the average nano-silica size. The surface area of nano silica is in the range of 175–225 m^2^/g with the density of 2.6 g/cm^3^. The obtained silica is stored and used for project purposes. It has been found to have around 98 per cent silica, which is extremely resistant to forming compound C–S–H. The SEM image of nano silica is presented in Fig. 1, showing the different particle size ranges of nano-silica from size 68.64 nm to size 152.32 nm. Figure 2 shows the EDX image, it represents high silica content in the nano silica.

Out of 8 sample specimens cast, 1 control specimen(CS) was cast, and replacement of the total mass of cement by fly ash by 20%, 40%, and 60% (F_20,_ F_40,_ F_60_) were done. Specimen cast with nano-silica (NS) is taken as reference and is the replacement of fly ash by 20%, 40%, and 60% (FN_20,_ FN_40,_ FN_60_) to the total mass of nano-silica replacement. Water Cement ratio was maintained as 0.35, superplasticizer was added below 0.5% to the mass of cementitious material by trial and error. In the concrete mixing machine, batches of concrete are produced by first adding water and rotating the machine for a few seconds to clean, followed by adding cement, fly ash, or nano-silica and coarse aggregate in dry form along with water and superplasticiser. All ingredients were mixed for 3 min followed by 3 min rest again it is mixed for 2 min finally.

## Experimental investigation

### Formwork and reinforcement

**Figure 3:**
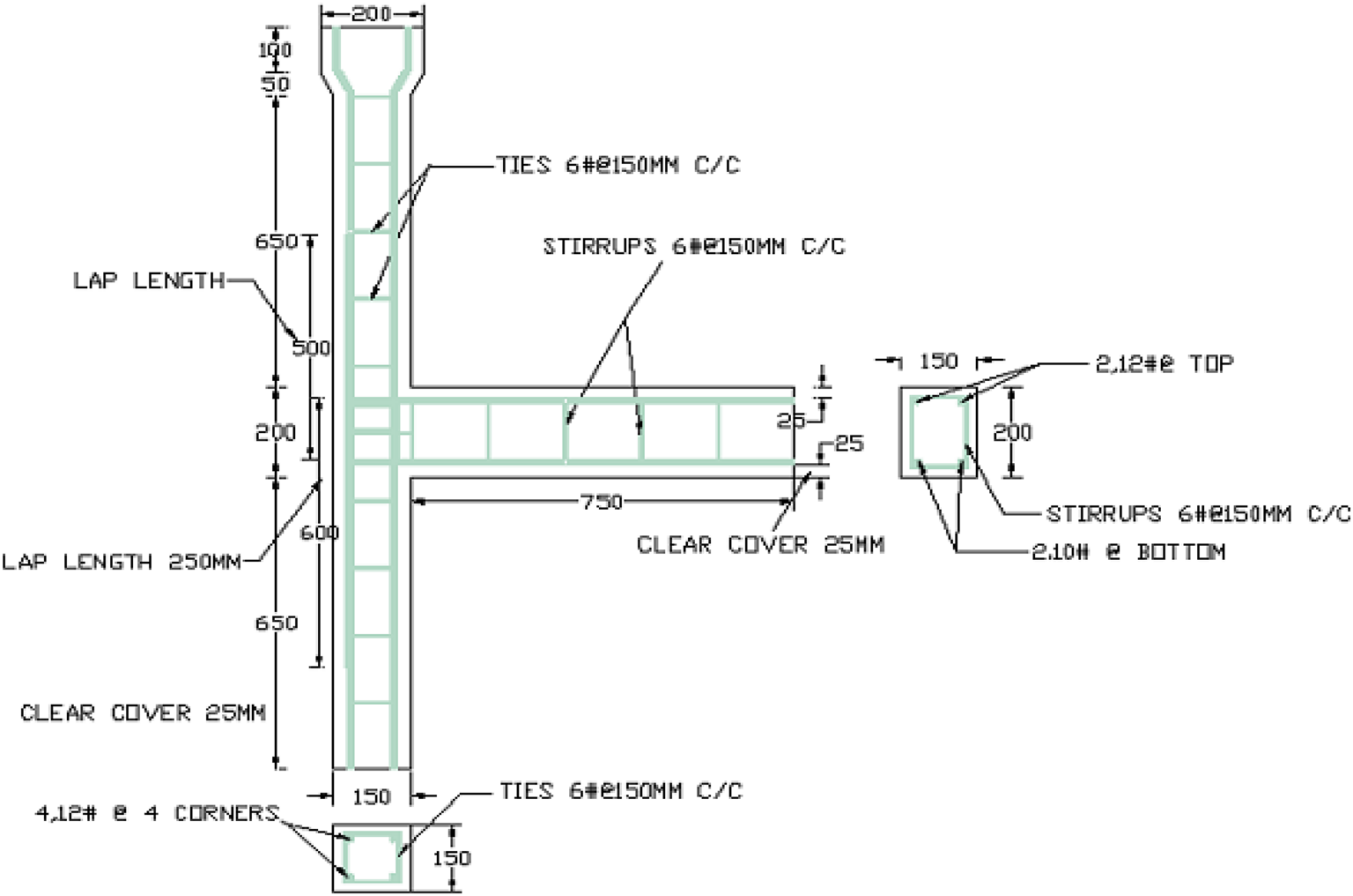
Formwork and reinforcement details.

**Figure 4:**
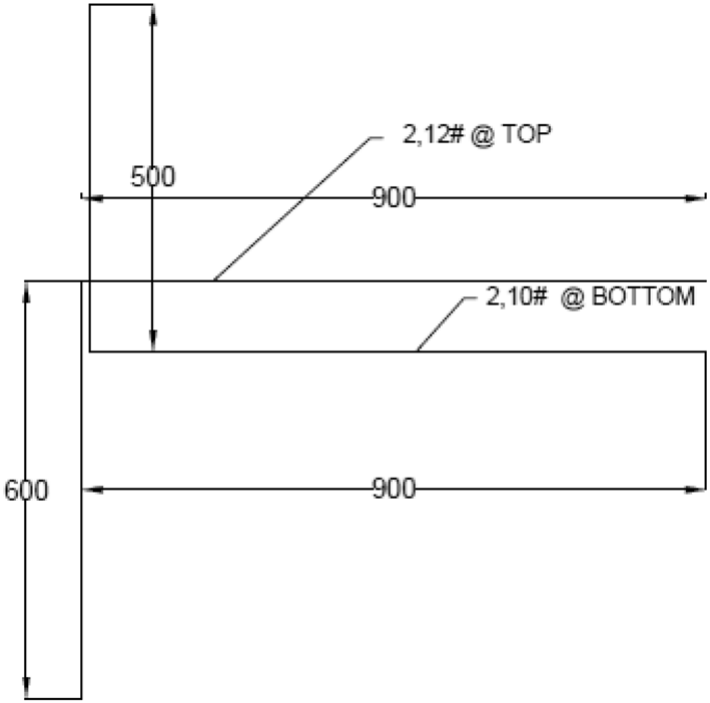
Seismic detailing of beam-column joint.

Brittle material properties, flexural strength, rupture module, bending strength, or fracture strength are defined as a material's ability to resist deformation under load. The flexural intensity shows the maximum stress experienced within the material during the breakup. The test specimen is cast and healed for 28 days, and the maximum load is tested. The compressive stress is maximal at the inside surface and minimum stress at the outside surface. These inner and outer edges are known as the 'extreme fibres' of the beam or rod. In this study, the samples produced are of column length—1650 mm, cross-section—150 mm × 150 mm, beams length—750 mm, cross-section—150 mm × 200 mm. Column head—100 × 200 mm. The specimen for the test was scaled to a 1/5 size fitting in the loading arrangement. The bending moment causes the beam to bend flexibly, while the axial deformation of the post-tensioned strand and mild steel bars causes the end cross-section of the beam adjacent to the column to rotate^31^. Although vertical bars prevented the column-joint interface from bending, failure occurred at the joint in this Investigation. Diagonal bars can also help to keep joints from failing^32^. Mild steel bars were used for Reinforcement, and Reinforcement details are shown in Figs. 3 and 4. The yield strength and ultimate strength of mild steel bars are 240 Mpa and 410 Mpa respectively. The column reinforcement is made with 4 rods of 12 mm diameter, and beam reinforcement is 2 rods of 12 mm diameter at compression zones and 2 rods of 10 mm diameter rods at tension. Stirrups of 6 mm diameter rod at 150 mm c/c were used in columns and beams. The specification of the test specimens (CS, F_20,_ F_40,_ F_60,_ NS_,_ FN_20,_ FN_40,_ FN_60_) is given in Fig. 4. The formwork was made according to the size of the beam-column joint and reinforcement details. Totally 8 specimens were cast and cured by covering them with rags and maintaining the moisture content. The proposition of mixes is provided below in Table 1.

**Table 1:** Proposition of mixes.

Mix ID	Binder (kg/m^3^)		Aggregates (kg/m^3^)		Nano silica	Water (kg/m^3^)
	Cement	Fly ash	Fine	Course		
CS	480	0	860	720	0	192
F_20_	384	96	860	720	0	192
F_40_	288	192	860	720	0	192
F_60_	192	288	860	720	0	192
NC	480	0	860	720	2.5%	192
FN_20_	384	96	860	720	2.5%	192
FN_40_	288	192	860	720	2.5%	192
FN_60_	192	288	860	720	2.5%	192

**Figure 5:**
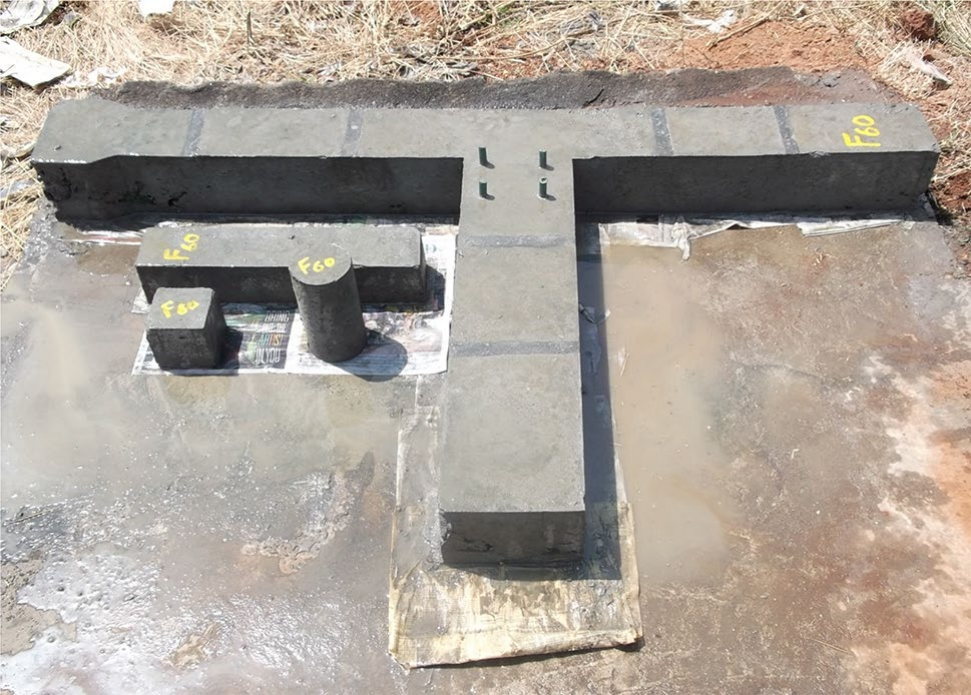
Cast specimen.

The axial load capacity generally exceeds 50–60% in the joints. The impact of lateral loading (seismic forces) is high. A maximum column's axial load of 200 kN was reached, which is lower than 50% of the capacity of the column. In the cantilever beam, the point load was applied at the end until the failure of the specimen occurred. Compared to compressive strength, longitudinal reinforcement ratio and lateral beam effect, the most significant parameters are the bar anchorage of the beam and joint vertical stirrups^28^. The specimen casted for testing is shown in the Fig. 5.

### Test setup and instrumentation

**Figure 6:**
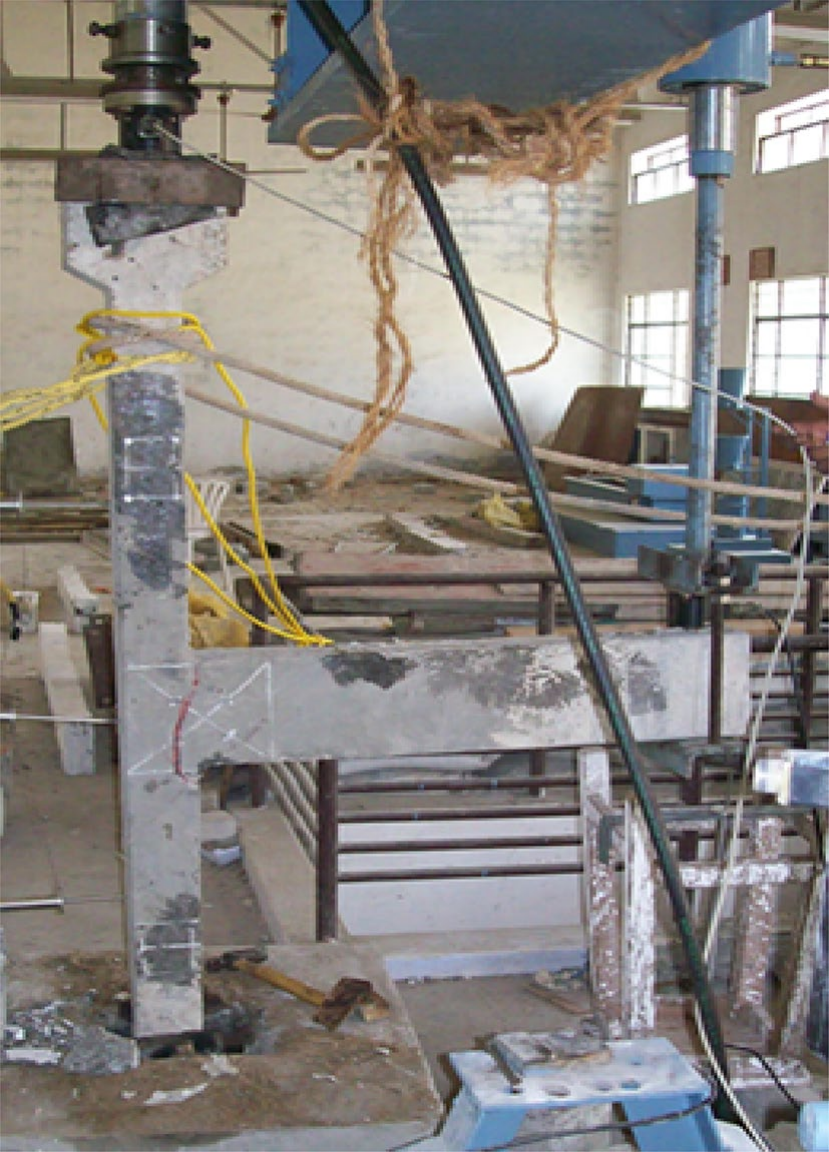
Testing arrangement for the samples.

**Figure 7:**
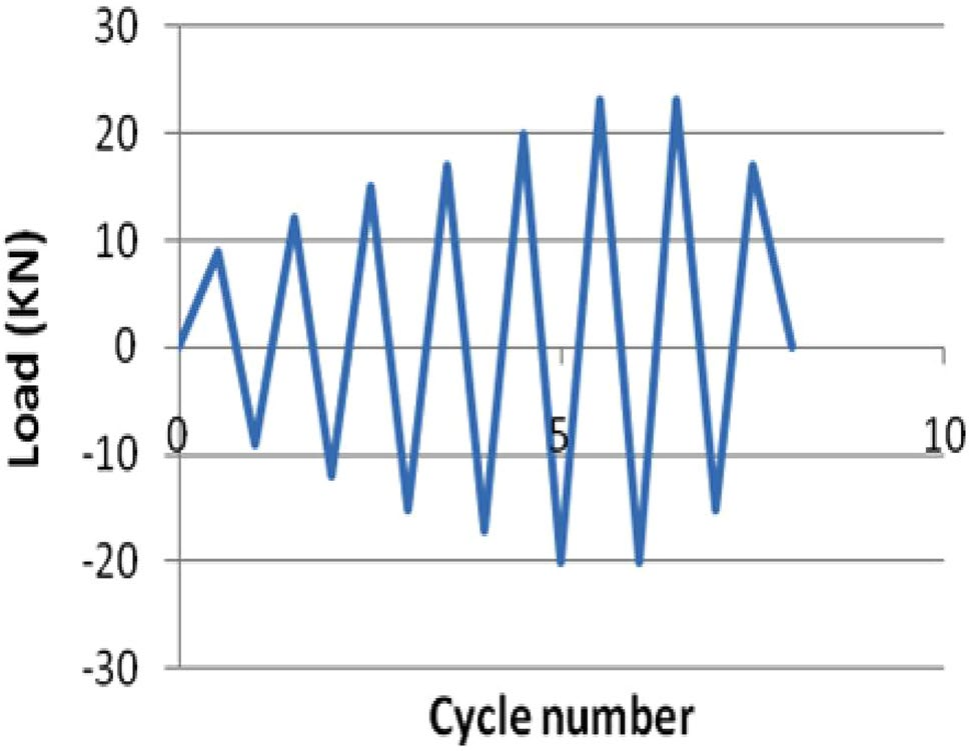
Load versus cycle number chart.

The samples produced were tested after curing for 28 days. The beam is positioned in the middle of the column in the cast specimen, as shown in Fig. 6. The column is fixed with the frame's loading frame, as two interior beam-column joints are selected for analysing the failure mechanism. The model designed by Breccolotti^21^ can investigate concrete's inelastic behaviour under monotonic, cyclic, or dynamic loads. It also enables the assessment of material stiffness degradation^21^. The frictional force at the point of contact between the beam and column flanges transmits the shear force created in the connection of the beam to the column^33^. The column is subjected to a 100 kN axial load, representing 50% of the column capacity. This implied the service load the section must convey under typical loading conditions. Due to the high strength and non-yielding of prestressing strands, the precast concrete connections had higher flexural strength in the positive direction and less severe stiffness degradation than the monolithic specimen^34^. A cyclic loading sequence with constantly increasing load and substantial inelastic excursions was chosen to draw inferences for the ultimate limit state and to test the performance under arbitrary seismic excitations^35^. Cyclic loading was applied to utilise a pressure-driven hydraulic actuator. Overall, cyclic loading tests give a comprehensive assessment of the behavior and performance of reinforced concrete beam-column connections under realistic and dynamic settings, enabling engineers design more durable and safe structures. The loading cycle was predefined, as appeared in Fig. 7. A total of 8 cycles of loading were used. The sample was benchmarked at the neutral position and swayed pleasingly about that position. Each cycle consists of 3 complete waves of identical amplitude in 10 s (0.3 Hz. frequency), increased at a consistent rate of 0.25 mm/cycle.

Increases in the axial compression ratio can improve the bearing capacity and initial stiffness of RC beam-column joints up to a certain point. Still, they can also reduce the deformation capacity and flexibility. The last tip redirection was 22.5 mm. Increases in the axial compression ratio below a particular value can greatly improve the bearing capacity and initial stiffness of RC beam-column joints. However, deformation capacity and flexibility will be reduced^36^. Vertical redirection of the tip of the column was noted down directly by the linear variable displacement transducer (LVDT) worked in the actuator. It was approved with another outside LVDT. The information was gathered utilizing a mechanized information procurement framework.

The typical load sequence diagram is shown (Fig. 6) below; if the specimens accomplish their strength limit, strength deprivation may occur due to large lateral displacement^22^. In the 3 cycles, the load attainment is nearly 16.5 kN, which shows 73.5%, nearly 75% of the peak load of 22.5 kN.

## Results and discussion

The strain experienced by reinforcement bars was observed using strain gauges. Yield strain occurred at joint zones, and plastic hinges were elastic. Adjacent to the joint zones in the column, the longitudinal Reinforcement is in the elastic limit.

**Figure 8:**
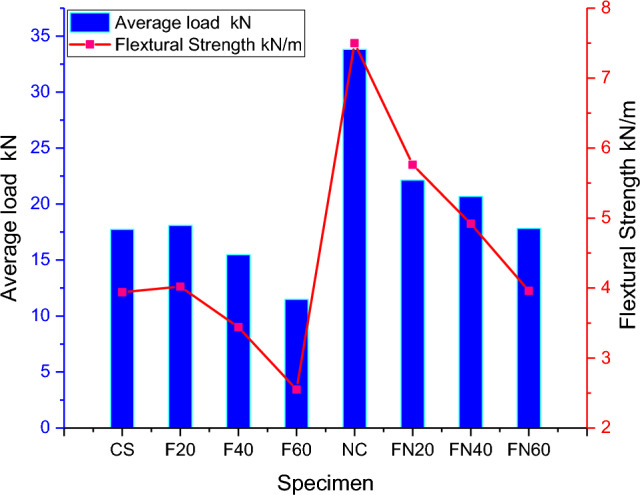
Flexural strength of the specimen.

**Figure 9:**
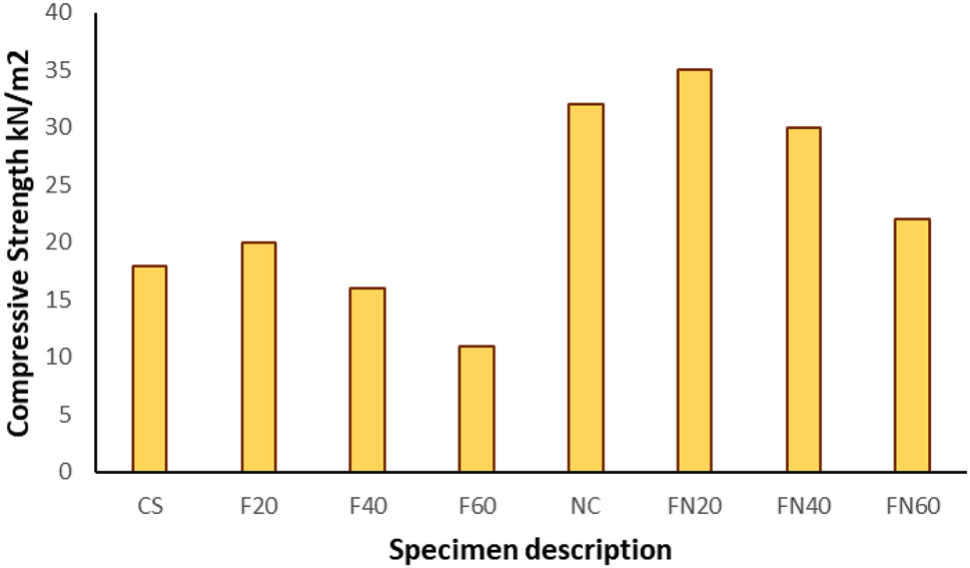
Compressive strength for the samples.

**Figure 10:**
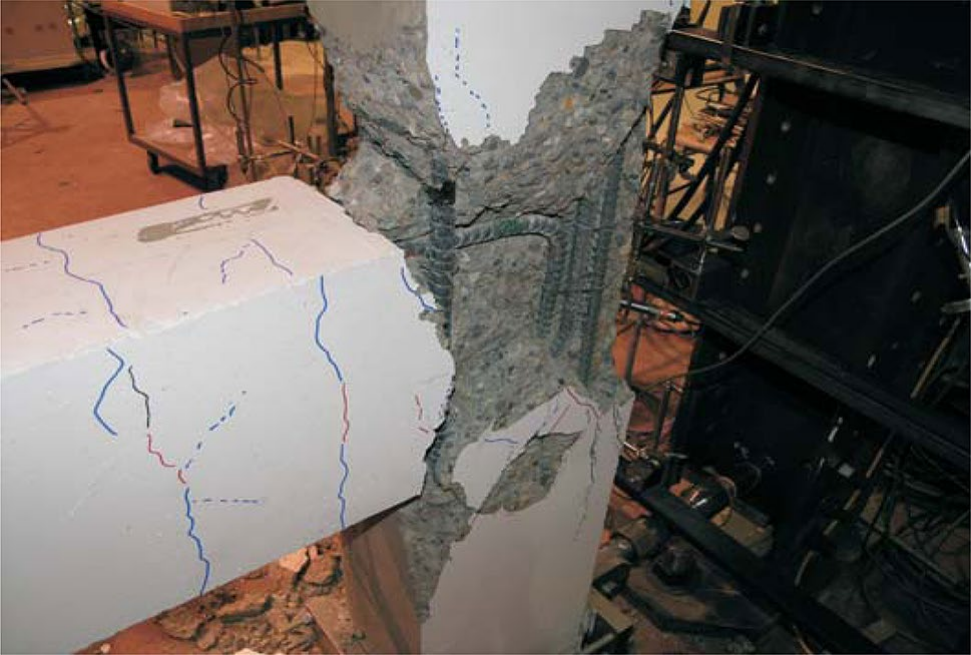
Failure of beam-column joint.

Until they fail under compressive stress, most materials fail under tensile stress, so the highest tensile stress can be maintained until the beam or rod fails is its flexural strength. Figures 8 and 9 shows the flexural Strength and compressive strength of the specimen respectively. High flexural strength was observed in the NC specimen, Fig. 10 represents the failure of Beam-Column Joint. Table 2 represents the end Deflection of member of the specimen.

**Table 2:** End deflection of member.

Specimen description	Max lateral load (kN)	Column axial load (kN)	Number of cycles	Max. tip deflection of beam (mm)
CS	15	60	15	4.52
F_20_	18	60	18	14.62
F_40_	12	60	12	10.18
F_60_	8	60	8	8.99
NC	35	60	35	43.85
FN_20_	27	60	27	37.54
FN_40_	21	60	21	18.81
FN_60_	19	60	19	10.44

The rectangular specimen under a load in three-point bending is given by

2 $$\sigma = 3{\text{FL}}/{\text{bd}}^{2} \left( {{\text{kN}}/{\text{m}}^{2} } \right)$$

where L—support span length, b—width, d—thickness, F—Load at the fracture point.

**Figure 11:**
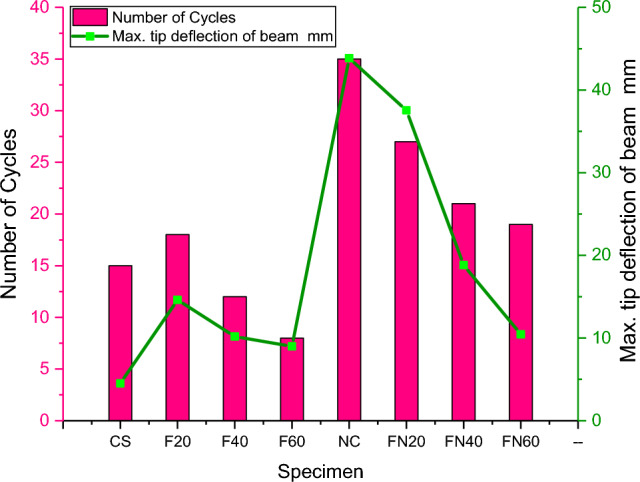
Maximum tip deflection of the beam.

**Figure 12:**
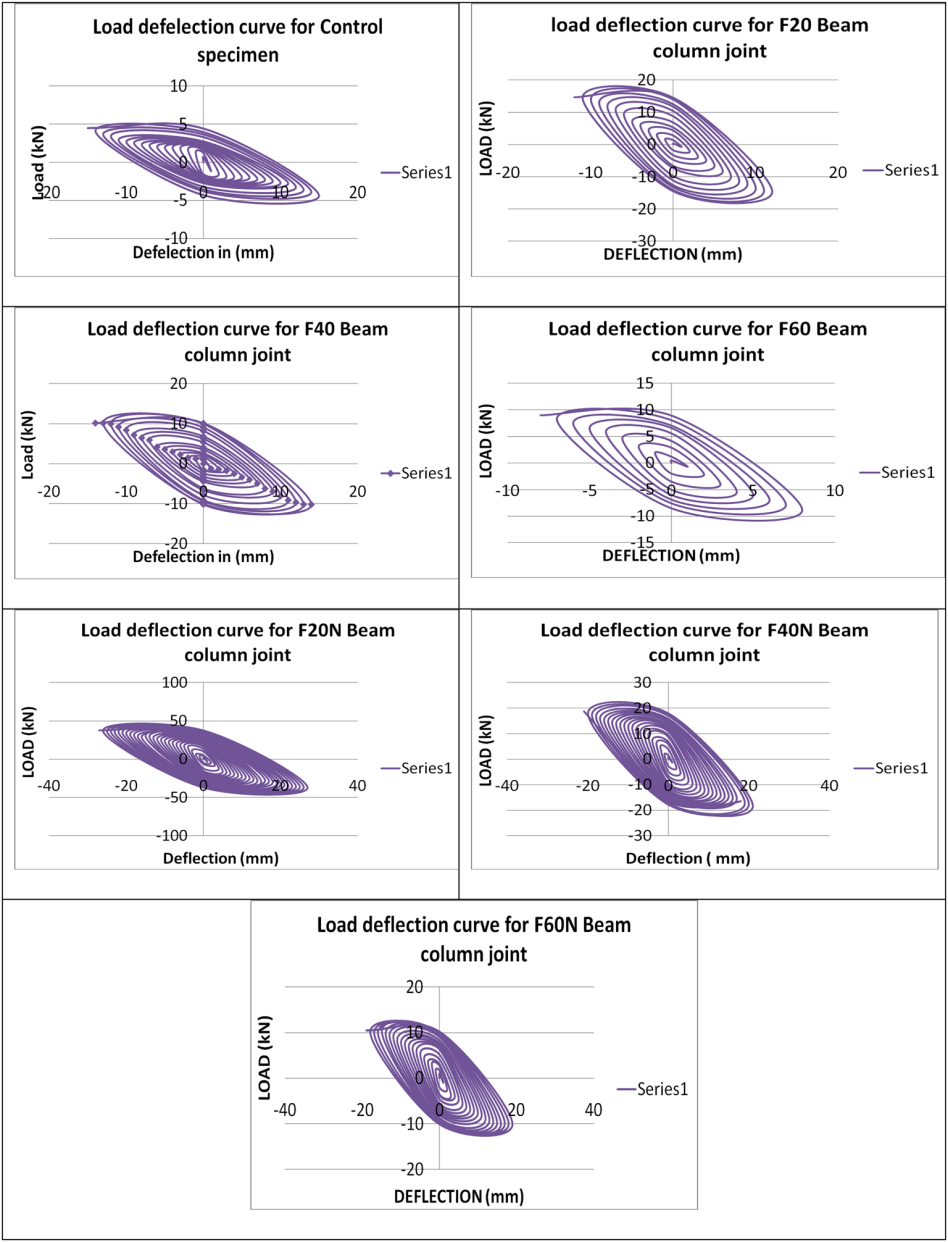
Load—deflection curve of the tested samples.

The critical joint in the structures can be analysed using the limit of energy distribution in the member. Some types of energy distribution include hysteretic damping, kinetic energy, and thick damping. The hysteretic damping can occur at regions encased in loops of displacement hysteresis. In two cases, the hysteretic circles were very wide, thus showing that a high energy scattering limit, soundly with the bendable failure component outwardly observed. Specifically, the column segment joint arranged with F20N and F40N concrete performed better at higher cycles, as shown in Figs. 11 and 12.

## Conclusion

The study explored the performance of nano silica beam-column joints exposed to cyclic loading. As the level of a fly is included in the increments in concrete, the flexural quality also elevates to a specific range of (20–40%), and afterwards, it diminishes. It was observed that a gradual increase in fly ash causes extended setting time and slow strength development, leading to low early-age strengths and delays in the rate of construction. From the practical observation, it is noted that the surge of fly ash reduced the workability of concrete. The increase of nano-silica achieves flexural quality and workability. The chart shows that FN20 and FN40, cement’s strength, are the most extreme. It is observed that FN 60 fails under a maximum lateral load of 19 kN, which is 4 kN greater than the controlled specimen.

Consequently, as the seismic burden increases, the tensile strength in steel is lower in the nano-silica composites than in the fly ash composites. Although seismic loads do not have a direct impact on the tensile strength of materials, they can induce cyclic loading in structures when earthquakes occur. The investigation of the response of materials, namely nano-silica and fly ash composites, to repeated loading is of utmost importance in guaranteeing the durability and security of structures located in areas susceptible to seismic activity. A comprehensive comprehension of the manner in which materials react to cyclic loads is crucial in the process of creating earthquake-resistant structures and infrastructure. The utilisation of these materials can accomplish a significant increment in yield burden. Since it has minimal wear and tear, we can suggest this cement for exceptional conditions, such as shelters, storehouses, petroleum tanks, and overwhelming industrial structures.

## Data Availability

The datasets used and/or analysed during the current study available from the corresponding author on reasonable request.
